# Mobile Health Interventions for Individuals with Type 2 Diabetes and Overweight or Obesity—A Systematic Review and Meta-Analysis

**DOI:** 10.3390/jfmk10030292

**Published:** 2025-07-29

**Authors:** Carlos Gomez-Garcia, Carol A. Maher, Borja Sañudo, Jose Manuel Jurado-Castro

**Affiliations:** 1 Physical Activity and Sport Sciences, University School of Osuna (Attached to the University of Seville), 41640 Osuna, Seville, Spain; carlosgg@euosuna.org (C.G.-G.); juradox@gmail.com (J.M.J.-C.); 2 Alliance for Research in Exercise, Nutrition and Activity, Allied Health and Human Performance, University of South Australia, City East Campus, GPO Box 2471, Adelaide, SA 5001, Australia; carol.maher@unisa.edu.au; 3 Department of Physical Education and Sport, University of Seville, 41013 Seville, Spain; 4 Metabolism and Investigation Unit, Maimonides Biomedical Research Institute of Cordoba (IMIBIC), Reina Sofia University Hospital, University of Cordoba, 14004 Cordoba, Spain; 5 CIBER Physiopathology of Obesity and Nutrition (CIBEROBN), Institute of Health Carlos III, 28029 Madrid, Spain

**Keywords:** chronic disease, diabesity, digital health, exercise, telemedicine

## Abstract

**Background**: Type 2 diabetes (T2D) and overweight or obesity are strongly associated, with a high prevalence of these concomitant conditions contributing significantly to global healthcare costs. Given this burden, there is an urgent need for effective interventions. Mobile health (mHealth) technologies represent a promising strategy to address both conditions simultaneously. **Objectives**: This systematic review and meta-analysis aimed to evaluate the effectiveness of mHealth-based interventions for the management of adults with T2D and overweight/obesity. Specifically, it assessed the quantitative impact of these interventions on glycosylated hemoglobin (HbA1c), body weight, triglycerides, total cholesterol, low-density lipoprotein (LDL), and high-density lipoprotein (HDL) levels. **Methods**: A systematic search was conducted in PubMed, Web of Science, and Scopus databases from inception to 9 July 2025. The inclusion criteria focused on randomized controlled trials (RCTs) using mHealth interventions in adults with T2D and overweight/obesity, reporting HbA1c or weight as primary or secondary outcomes. The risk of bias was assessed using the Cochrane Risk of Bias tool 2. A total of 13 RCTs met the inclusion criteria. **Results**: Meta-analysis indicated significant improvements after 6–12 months of intervention in HbA1c (MD −0.23; 95% CI −0.36 to −0.10; *p* < 0.001; I^2^ = 72%), body weight (MD −2.47 kg; 95% CI −3.69 to −1.24; *p* < 0.001; I^2^ = 79%), total cholesterol (MD −0.23; 95% CI −0.39 to −0.07; *p* = 0.004; I^2^ = 0%), and LDL (MD −0.27; 95% CI −0.42 to −0.12; *p* < 0.001; I^2^ = 0%). **Conclusions**: mHealth interventions are effective and scalable for managing T2D and obesity, particularly when incorporating wearable technologies to improve adherence. Future research should focus on optimizing personalization, engagement strategies, and long-term implementation.

## 1. Introduction

Diabetes mellitus (DM) stands as one of the most prevalent chronic diseases globally [1], with an estimated 422 million adults affected in 2014 compared to 108 million in 1980. The global prevalence of DM nearly doubled between 1980 and 2014, increasing from 4.7% to 8.5% in adults [1]. There is a strong association between DM and obesity [2], with obesity acting as a major risk factor for developing type 2 diabetes (T2D) by contributing to insulin resistance and placing excess demand on the pancreas, potentially leading to beta-cell dysfunction [3]. Approximately 90% of T2D cases are linked to excess weight, and 197 million people are estimated to have impaired glucose tolerance, largely due to obesity and metabolic syndrome—a figure expected to reach 20 million by 2025 [4]. The economic burden of T2D and obesity is substantial, with diabetes complications alone accounting for 14–15% of healthcare costs in many countries [5].

Given the high prevalence and cost of these comorbid conditions, effective prevention strategies are essential, with weight loss being a fundamental component [6]. Extensive research has established the importance of improving physical activity (PA) and dietary habits in preventing or delaying the onset of T2D, as well as reducing cardiovascular risk [7,8]. Landmark studies, such as the U.S. Diabetes Prevention Program (DPP), have shown that modest weight loss (5–7%) through intensive lifestyle support can reduce diabetes risk by up to 58%, with each kilogram lost corresponding to a 16% risk reduction [9]. Notably, participants who met PA goals—even without achieving the weight loss target—had a 44% reduced incidence of diabetes [9].

PA has been shown to yield benefits equivalent to weight loss in many cases. Regular physical activity is associated with improved glycemic control, enhanced insulin sensitivity, reduced cardiovascular and microvascular complications, improved mental health, and better quality of life and cognitive function, particularly in older adults [7,10,11,12,13,14,15].

However, physical inactivity remains a critical concern. Defined as performing less than 150 min of moderate-intensity aerobic activity per week, aerobic inactivity is now the fourth leading cause of death and contributes to over 40 chronic diseases, including T2D and obesity [16,17,18]. Recent estimates show that 31% of adults do not meet recommended PA levels [17,19], a figure confirmed by multiple large-scale surveys [17]. These trends highlight the urgent need for scalable, effective interventions to promote PA at the population level [20].

Traditional lifestyle interventions typically involve in-person sessions, educational materials, and phone support. While effective in the short term, these strategies are resource-intensive and often fail to achieve sustained behavioral change [21,22]. In this context, mobile health (mHealth) interventions—delivered via smartphones, tablets, wearables, and other digital platforms—have emerged as a promising, scalable solution [23,24,25,26,27,28,29].

Although mHealth strategies have shown positive effects in managing T2D or obesity separately, there is a notable lack of evidence assessing their effectiveness in individuals affected by both conditions simultaneously. This is a critical gap, as T2D and obesity often coexist and interact in ways that may attenuate the effectiveness of behavioral interventions. For example, severe insulin resistance, chronic inflammation, and reduced physical capacity in this population may diminish the benefits of physical activity on glycemic control [10]. Furthermore, comorbidities and differing metabolic responses may alter adherence, outcomes, and treatment needs.

Previous systematic reviews have generally evaluated mHealth interventions targeting T2D or obesity in isolation [30,31,32,33,34], without exploring their joint impact. Some reviews have described app development and usability among individuals with chronic diseases [23,29,30,31,32,35,36,37,38,39,40,41,42,43,44,45], but they have not adequately addressed intervention effectiveness in the dual-diagnosis population. Moreover, methodological shortcomings—such as the inclusion of non-clinical studies, a lack of quantitative synthesis, and a limited review scope—further restrict the utility of prior findings [29,43].

A systematic review that specifically addresses individuals concurrently living with T2D and overweight/obesity could yield clinically meaningful insights. By analyzing this subgroup, we can better understand the unique barriers, physiological responses, and intervention outcomes relevant to their care. This knowledge is crucial to informing the development of more targeted and effective mHealth strategies for real-world implementation. Therefore, the aim of the present systematic review and meta-analysis is to evaluate the effectiveness of mHealth interventions in managing concurrent T2D and obesity, with particular focus on their impact on key clinical outcomes such as HbA1c, body weight, and lipid profiles.

## 2. Materials and Methods

The reporting of this systematic review was guided by the standards of the Preferred Reporting Items for Systematic Review and Meta-Analysis (PRISMA) [46] (Table S1). Furthermore, the revision protocol was registered in advance in the International Prospective Register of Systematic Reviews (PROSPERO) with ID CRD42024497708.

### 2.1. The Criteria for Considering Studies for Inclusion in the Review

Randomized controlled trials (RCTs) published between 2010 to 9 July 2025 were included in this systematic review and meta-analysis. Limitations on language were not applied in this study.

Inclusion criteria were established according to the PICOS strategy for the research question. “Population” (P): adults patients with T2D and overweight or obesity; “Intervention” (I): using mobile technology (mHealth), wearable activity tracker, mobile-based support or coaching, and training or awareness meetings about dietary habits, PA, or self-monitoring; “Control” (C): no intervention, usual care, waiting list control, or similar interventions without mHealth component; “Outcomes” (O): studies were required to include either glycosylated hemoglobin (HbA1c) or weight as a primary or secondary outcome; and the “Study Design” (S): RCTs.

Studies were excluded if they enrolled participants with type 1 diabetes, did not report outcomes on weight loss, or were reviews or meta-analyses. Studies were also excluded if mobile apps were simply used for communication between patients and health care providers.

The following studies were excluded: studies that included participants with diseases that relevantly affect glucose metabolism (e.g., hypertension); studies with patients affected by other particular situations (e.g., pregnancy or postpartum); and studies where the COVID-19 pandemic situation substantially impacted the results.

### 2.2. Protocol for Electronic Searching

The bibliographic search was carried out through the PubMed, Web of Science (WOS), and SCOPUS electronic databases.

Two different searches were conducted, first by using the following terms: “Diabetes” AND “Overweight” OR “Obesity” OR “Metabolic Syndrome”. Secondly, the terms “mhealth” OR “wearable” OR “apps interventions” OR “mobile apps” were used. Finally, both searches were linked with the Boolean operator “AND”. More details about terms included in the electronic search are shown in the Search Strategy (Supplementary File S1). All identified papers were critically assessed in order to choose those that met the proposed criteria.

The results of the systematic literature search were extended by a snowball principle. For this purpose, the references of relevant and included articles were screened with backward snowballing and forward snowballing using CitationChaser (https://estech.shinyapps.io/citationchaser/, accessed on 10 July 2025). The same assessment procedure with title, abstract, and full-text screening was conducted.

### 2.3. Study Selection and Data Extraction

Two researchers (C.G.-G. and J.M.J.-C.) conducted the literature search and undertook study selection and data extraction independently. The bibliography search was performed in two phases. During the first phase, papers were screened based on their title and abstract. Articles that did not meet the inclusion criteria were excluded. In the second phase, the full text the remaining articles was carefully read, and eligible studies proceeded to data extraction. Information and data about the characteristics of the population, the type of intervention, and the main results of the studies were extracted.

The articles were organized using the reference manager software Mendeley 1.19.8 (USA production), with discrepancies regarding study selection and extracted data discussed at each stage. Any unresolved disagreements were resolved in a consensus meeting between the independent reviewers with a third reviewer (B.S.).

### 2.4. Risk of Bias in Individual Studies

The risk of bias in the included studies was assessed using the RoB 2.0 tool (Risk of Bias 2.0), recommended by the Cochrane Collaboration for randomized controlled trials [47]. The assessment considered the five standard domains: (1) bias arising from the randomization process, (2) bias due to deviations from intended interventions, (3) bias due to missing outcome data, (4) bias in the measurement of the outcome, and (5) bias in the selection of the reported result.

For each domain, as well as for the overall assessment, the standard judgments were applied: low risk of bias, some concerns, or high risk of bias. No strict thresholds or cut-off points were applied during the risk of bias assessment. All studies were retained regardless of their risk level to ensure a comprehensive review of the available evidence. However, the risk of bias ratings were considered when interpreting the findings, and sensitivity analyses were planned to assess the potential impact of study quality on the conclusions.

The evaluation process was conducted independently by two reviewers (C.G.-G. and J.M.J.-C.), both experienced in systematic reviews. Discrepancies were resolved through discussion, and when necessary, by consultation with a third reviewer (B.S.). The consensus was documented for each case.

The results of the risk of bias assessment are presented in the Results section.

### 2.5. Quality of Evidence

Overall qualitative analysis was performed using the Grading of Recommendations, Assessment, Development, and Evaluation (GRADE) system (https://www.gradepro.org, accessed on 12 July 2025), based on five domains: study design, risk of bias, inconsistency, indirectness, and imprecision [48].

After this, evidence was classified as follows: high quality, expressing confidence that the actual effect is close to the estimated one; moderate quality, conveying that the actual effect is likely to be close to the estimated one, but could be substantially different; low quality, meaning that the true effect could be substantially different; and very low quality, communicating that the actual effect is likely to be substantially different [49]. These evaluations were conducted by two researchers (C.G.-G. and J.M.J.-C.).

A qualitative analysis was conducted to summarize the quality of the evidence regarding the effectiveness of mHealth interventions in managing the main study variables (HbA1c and body weight) in adults with type 2 diabetes and overweight or obesity (Table 1), demonstrating moderate-quality evidence for both variables.

### 2.6. Statistical Analysis

All statistical analyses were performed using the software Review Manager (RevMan, Version 5.4, The Cochrane Collaboration, 2020) [50].

If the Standard Deviation (SD) of the post–pre differences in the experimental groups (EGs) and control groups (CGs) were not reported in the selected studies, then it was calculated from confidence intervals (CIs), standard error, or *p*-value of the absolute change of the different outcomes using standardized formulae [51]. If none of these data were available, the following formula was employed:

$$\mathrm{SD}\;=\;\sqrt{{\mathrm{SD}}_{\mathrm{pre}}^{2}+{\mathrm{SD}}_{\mathrm{post}}^{2}-(2\times r\times {\mathrm{SD}}_{\mathrm{pre}}\times {\mathrm{SD}}_{\mathrm{post}})}$$

where *r* is the correlation coefficient that describes how similar the pre- and post-measurements were across participants [51].

The effect of interventions on the different outcomes was analyzed by comparing the change in the EGs with the change in the CGs that did not receive intervention through mHealth. Data were obtained using the mean difference (MD) and SD of assessment data (numerical values) shown after the intervention at different moments: less than one month, at 3 months, and between 6 and 12 months, depending on the intervention’s duration.

The results of this meta-analysis are shown as a “forest-plot” with the MD and 95% confidence interval (CI). Heterogeneity is also presented and was calculated by measuring its extent by the I^2^ index. The *p*-value for this statistic was examined, noting the presence of heterogeneity when *p* < 0.05, which compromised the validity of the pooled estimates [52]. Furthermore, the I^2^ index of heterogeneity was considered low when values were between 0% and 40%; moderate between 30% and 60%; considerable between 50% and 90%; and substantial between 75% and 100% [51]. Furthermore, due to the presumed heterogeneity of the population and interventions in this study, a random-effects model was employed to measure the effect of the included studies [53]. Subgroup analyses were performed according to the duration of the intervention to examine its effect on the selected outcomes. Six studies [54,55,56,57,58,59] included in this meta-analysis performed multi-arm interventions that were included following the Cochrane guide [60].

## 3. Results

### 3.1. Studies Selected

**Figure 1:**
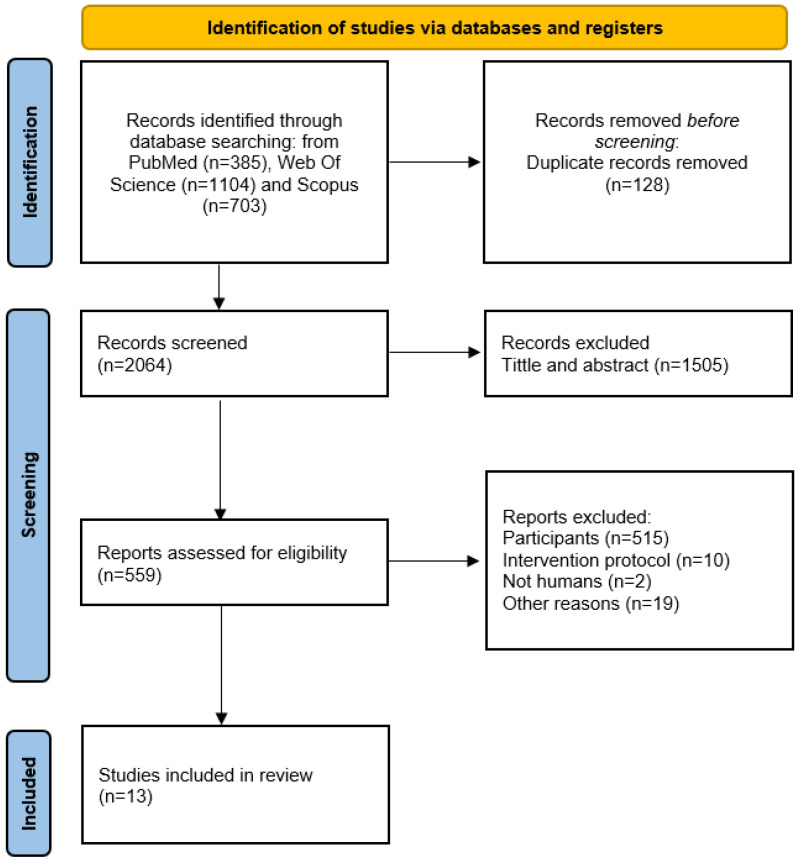
A flow chart showing the results of the selection process.

A flowchart diagram illustrates the selection of articles included in this meta-analysis (Figure 1). Initially, 2192 papers were identified from the various databases. After removing 128 duplicates, 2064 unique papers remained for potential inclusion. Of these, 1505 articles were excluded based on titles or abstracts that did not align with this study’s aims. This left 559 full-text articles that were assessed for eligibility according to the inclusion criteria. However, 546 of these were excluded for not meeting the inclusion criteria. Ultimately, thirteen articles were included in the present systematic review and meta-analysis.

### 3.2. Description of Selected Studies

The characteristics of participants, dropout, baseline data, and outcomes of the eligible trials of the meta-analysis are detailed below (Table 2). Across all studies, there was a total of 1928 (EG, n = 1061; CG, n = 867; 52% males; 53.4 ± 10.1 years old) participants with T2D and overweight or obesity. Three studies [57,59,61] involved prediabetic participants (% Hb1Ac = 5.8 ± 1) and the rest of the studies ranged between 6.5 and 9.2%. The Body Mass Index (BMI) range was 26.5–39.4 kg/m^2^.

Five studies addressed Hb1Ac (%) as the primary outcome [57,58,62,63,64]; five studies addressed weight (kg) as the primary outcome [55,56,59,61,65]. Four studies addressed Hb1Ac, weight, and the rest of the outcomes included in the review as secondary outcomes.

All studies used a mobile app or similar software in their interventions, but four studies [54,57,63,66] out of the thirteen studies also used a wearable in their interventions (Table 3). To support self-management of their conditions, some studies provided participants with various devices: Bender et al. [54] used an accelerometer, a step counter, and a calorie intake tracking app. Bentley et al. [66] provided a wearable device for automatically recording PA. De Luca et al. [63] employed a glucometer, a sphygmomanometer, bodyweight scales, a smartwatch for heart rate monitoring, and a step counter. Kim et al. [67] used a step counter. Wang et al. [57] used pedometers, weight scales, and food scales. Yin et al. [58] did not use wearable devices but provided glucometers. Furthermore, some studies [57,58,59,63] were focused on how self-monitoring (PA minutes a week or daily steps, calorie intake, glycosylated hemoglobin, or weight) could help to improve the treatment of pathologies. The rest of the studies [54,55,56,61,62,64,65,66] were focused on how telehealth coaching through a mobile app could impact the treatment compared with usual care. Overall, app-based coaching sessions were focused on lifestyle (physical activity, diet, sleep, and stress). A hybrid intervention (a technology-based intervention combined with conventional care) was used in four studies. In terms of the control condition, two studies [61,66] gave some advice on diet and exercise to their CG during the intervention process, another study [54] received a Fitbit, while all other studies involved usual care control groups.

The characteristics of interventions of the selected studies are described below (Table 3). The total duration of the intervention for two studies [66,67] was 3 months, for eight studies [54,57,58,59,62,64,65,67] it was 6 months, for one study [63] it was 8 months, for one study [56] it was 12 months, and in the final study it was 24 months [55].

No adverse events were reported in any studies.

**Table 2:** Characteristics of participants, % dropouts, baseline data, and outcomes of the eligible trials included in the meta-analysis.

Study (Year)	Setting	Total (EG/CG)	% Males	Age (Years), Mean (SD)	EG/CG Pre-Intervention	EG/CG Post Intervention	% Dropout	% HbA1c at Baseline or Range (mmol/mol)	BMI (kg/m^2^) at Baseline or Range	Primary Outcomes	Secondary Outcomes
Bender et al. (2017) [54]	USA	n = 45 (EG, n = 22/CG, n = 23)	38%	57.6 (9.8)	n = 45 (22/23)	n = 45 (22/23)	0%	7.42 (0.8)	30.1 (4.6)	Adherence to additional mHealth engagement measures	Weight, BMI, HbA1c, and daily step counts.
Bentley et al. (2016) [66]	UK	n = 27 (EG, n = 18/CG, n = 9)	44%	52.9 (8.6)	n = 27 (18/9)	n = 20 (13/7)	25%	Range between 57.6 and 65.8	Range between 25 and 40	Adherence to using the device	Wright and HbA1c
Block et al. (2015) [62]	USA	n = 339 (EG, n = 163/CG, n = 176)	69%	55.0 (8.9)	n = 339 (163/176)	n = 292 (136/156)	13%	5.6 (0.3)	31.2 (4.4)	HbA1c and fasting glucose.	Weight, BMI, waist circumference, TG to HDL ratio, and metabolic syndrome.
Christensen et al. (2022) [55]	Denmark	n = 340 (EG, n = 200/CG, n = 140) with T2D n = 168 (EG, n = 98/CG, n = 70)	With 24 months follow up: 38% Without 24 months follow up 35%	With 24 months follow-up: EG: 53.9 (9.2) CG: 53 (11.6) Without 24 months follow-up EG: 51 (10.9) CG: 51.7 (12.1)	n = 340 (200/140) with T2D n = 168 (98/70)	n = 132 (81/51) with T2D n = 65 (40/25)	61% total participants	Range between 47.6 and 48.9	34.7 (3.9), 35.7 (3.8), 35.6 (3.7), 35.8 (5.0)	Weight	HbA1c level, waist/hip ratio (WHR), systolic and diastolic blood pressure, total TG, HDL, LDL, smoking status, and quality of life.
Christensen et al. (2022) [65]	Denmark	n = 170 (EG, n = 100/CG, n = 70)	EG 51%/CG 68%	EG 56.1 (7.3); CG 57.1 (9.9)	n = 170 (100/70)	n = 128 (75/53)	24%	7.4 (1.3)	34.7 (3.29), 35.0 (4.40)	Weight	HbA1c
De Luca et al. (2023) [63]	Italy	n = 200 (EG, n = 100/CG, n = 100)	EG 83% CG 70%	EG 61.1 (9.4); CG 66.5 (9.0)	n = 200 (100/100)	n = 192 (92/100)	Not reported	7 (0.9)	29.6 (5.0)	Hb1Ac	Weight, blood pressure (systolic and diastolic), plasma cholesterol, plasma triglycerides, LDL, and HDL
Hesseldal at al (2022) [56]	Denmark	n = 338 (EG, n = 198/CG, n = 140)	37%	52.3 (11.0)	n = 338 (198/140)	n = 200 (127/73)	40%	6.6 (1.3)	35.3 (3.8)	Weight	HbA1c
Kim et al. (2024) [67]	Korea	n = 200 (EG, n = 134/CG, n = 66)	69%	EG 57.1 (7.2) CG 58.3 (5.8)	n = 200 (134/66)	n = 182 (119/63)	17%	7.1 (0.4)	26.5 (2.6)	Step counts	HbA1c
Lim et al. (2022) [59]	Singapore	n = 148 (EG, n = 72/CG, n = 76)	60%	53.1 (9.3)	n = 148 (72/76)	n = 140 (67/73)	5%	5.9 (0.5)	29.8 (4.1)	Weight (6 months)	HbA1c, FBG, blood pressure, serum lipids, creatinine, dietary intake and physical activity
Moravcová et al. (2024) [61]	Czech Republic	n = 100 (EG, n = 50/CG, n = 50)	29%	43.3 (9.5)	n = 84 (42/42)	n = 60 (32/28)	40%	5.7 (0.8)	39.4 (6.8)	Weight	HbA1c, BMI, waist circumference, body fat, fasting glucose
Wang et al. (2018) [57]	USA	n = 26 (EG1, n = 11/EG2, n = 9/CG, n = 6)	42%	56.4	n = 26 (11/9/6)	n = 24 (10/8/6)	7%	8.4% (2.3)–10.4% (2.4)	38.1 kg/m^2^	HbA1c	Weight
Whittemore at al (2020) [64]	Mexico	n = 47 (EG, n = 26/CG, n = 21)	35%	EG 53.9 (9.2) CG 56.8 (8.3)	n = 47 (EG, n = 26/CG, n = 21)	n = 44 (EG, n = 24/CG, n = 20)	6%	9.2% (1.5)	EG: 31.0 (6.1) CG: 29.5 (5.0)	HbA1c	BMI, diastolic and systolic blood pressure, and PA
Yin et al. (2022) [58]	China	n = 120 (EG, n = 60/CG, n = 60)	40%	47.3	n = 120 (60/60)	n = 99 (52/47)	17%	8.5% (0.8)	EC: 29.05 kg/m^2^ (3.31); CG: 29.2 kg/m^2^ (2.9)	HbA1c	Postprandial blood glucose, FBG, BMI, total cholesterol, TG, LDL and HDL, blood urea nitrogen, creatinine, and estimated glomerular filtration rate

BMI = Body Mass Index, CG = control group, EG = experimental group, FBG = fasting blood glucose, Hb1Ac = glycosylated hemoglobin, HDL = high density lipoprotein, LDL = low density lipoprotein, PA = physical activity, T2D = type 2 diabetes, TG = triglycerides.

**Table 3:** Characteristics of the interventions, type of technology used, duration, follow-up, adherence, tools, and observations of the eligible trials of the meta-analysis.

Study (Year)	Type of Intervention	Type of Technology Used/mHealth Tools Needed in the Intervention	Groups Description	Hybrid Intervention *	Duration of Intervention	Follow-Up	Adherence (%)	Observations
Bender et al. (2017) [54]	Lifestyle intervention based on diabetes prevention program, modified to incorporate mobile technologies (Fitbit accelerometer plus app with diary) and private Facebook group for healthy behaviors tracking, real-time feedback, coaching, and virtual social support.	Mobile-based, virtual support and Fitbit accelerometer/wearable plus associated mobile app	EG: Phase 1: (3 months): Self-monitor real-time PA steps and daily food/calorie intake, and weekly weight. Virtual social support, coaching, weekly education topics, and individualized weight loss goals. Phase 2: Transitioned to a 3-month follow-up, removed from private Facebook group, and asked to continue using their Fitbit and app with diary to track health behaviors and maintain weight goals. CG: The control group was a waitlist group. Phase 1: Received only the Fitbit accelerometer and training about daily wear. Phase 2: At the 3-month office visit, they transitioned to receive the PilAm Go4health Intervention.	No	6 months	EG: monthly for phase 1 Months 4 and 6 for phase 2 CG: months 1 and 3 for phase 1 Months 4, 5 and 6 for phase 2	Attendance to all visits: EG, 95%; CG, 100% Wearing the Fitbit at least 5 days/week: EG, 97%; CG, 91%	N/A
Bentley et al. (2016) [66]	Training on appropriate behaviors to lose weight and control HbA1c that included automatic recording of PA and nutritional intake for eating healthily by using a wearable device called AiperMotion 500 plus qualitative interviews.	Mobile-based, wearable device, email support service/wearable plus associated mobile app	EG: Divided into groups 2 and 3: both groups received 90 min group training around appropriate behaviors to lose weight and control their HbA1c, specifically: diet, maximizing PA, and neuro-linguistic programming. Group 2 received additional 60 min training in the use of the AiperMotion 500. They were asked to enter individual characteristics and dietary information. They were asked to wear the device during walking hours. They received motivational feedback. Group 3 was asked to send weekly emails to the research team describing any positive or negative events that had impacted their conformance with the study or motivation to lose weight. CG: Received 90 min group training around appropriate behaviors to lose weight and control their HbA1c, specifically: diet, maximizing PA and Neuro-Linguistic Programming but no further training on how to use the device. Impossible to download the data from the device (no feedback)	Yes	3 months	4 months	% days worn from total Weeks 1–6 (G2: 62%, G3: 61%); weeks 7–12 (G2: 65%, G3: 69%); weeks 13–16 (G2: 75%, G3: 94%). % diet entries (at least 950 kcal) from total Weeks 1–6 (G2: 62%, G3:61%); weeks 7–12 (G2: 59%, G3: 70%); weeks 13–16 (G2: 49%, G3: 70%). % emails asked (G3): 31%	N/A
Block et al. (2015) [62]	Alive-PD (program design). Alive-PD provides a 1-year program of regular contact and goal setting, weekly in the first 6 months and biweekly thereafter, plus midweek automated email and mobile phone reminders. The program includes individually tailored weekly goal setting and other activities delivered via web and email, supplemented by automated IVR phone calls and a supportive mobile phone app.	Mobile-based, web-based, and email supplemented by automated IVR phone calls/mobile app	EG: Received the Alive-PD. CG: No mobile-based interventions, emails, or phone calls were provided to this group. Participants continued receiving their usual care through the health center. They received no further contact from the online Alive-PD system except reminders to complete a 3-month and 6-month online follow-up questionnaire.	No	6 months	3 and 6 months, optional additional clinic visits at 9 and 12	EG, n = 163 set behavioral goals or otherwise interacted with the online Alive-PD in a median of 17 of the 24 weeks (70.8% of the weeks). In all, 87.1% (142/163) interacted with the program in 4 or more of the 24 weeks, and 70.6% (115/163) were still interacting with the program in the last month of the 6-month period.	N/A
Christensen et al. (2022) [55]	Telehealth lifestyle-coaching program (Liva 1.0) leads to long-term (24 months) weight loss compared to usual care.	Mobile-based and web-based telehealth lifestyle coaching program/mobile app	EG: Intervention using the Liva app telehealth lifestyle–coaching, starting with online face-to-face consultation to define SMART goals. After the first session, coaching was performed asynchronously. The first 6 months of structured educational material and motivational support were provided weekly from the lifestyle coaches, biweekly for the next 6 months, and after 12 months, participants only received structured educational material and lifestyle coaching every third month. CG: Participants randomized to the control group were offered to receive the standard municipal secondary or tertiary preventive care service with information about diet and physical activity, and how to develop healthy habits. A few of them included the promotion of well-being and social participation. The participants in the control group were not offered a specific ‘usual care’ program but participated in whatever the local municipality offered in accordance with the Danish Health Care Act of 2005.	No	24 months	6, 12, and 24 months	Not reported	Most of the dropouts were random or due to coronavirus disease 2019 restrictions
Christensen et al. (2022) [65]	eHealth app LIVA 2.0 (long-term lifestyle change intervention and eHealth application) combined with BCTs such as self-monitoring, reminders, tailored information, personal feedback, and face-to-face support.	Mobile-based and health coaching/mobile app	EG: They received the individualized digital lifestyle coaching LIVA 2.0. Each patient and their health coach discussed and agreed on goals for diet, physical exercise, sleep, and any other relevant lifestyle areas. Weekly coaching for the first three months, and biweekly for the next three months. The intervention included a high degree of BCTs. CG: Examinations at the same frequency as the intervention group. At the first examination, and after they were randomized in the control group, they were advised to contact their general practitioner (GP) who could provide guidance about their health problems and further refer them to diabetes programs in their municipalities that included education about diet, exercise, and different forms of behavioral change techniques (BCTs). The control group did not have access to the app, nor did they receive any digital interventions from LIVA 2.0.	Yes	6 months	6 months	Not reported	25% of patients lost to follow-up at six months due to unknown reasons
De Luca et al. (2017) [63]	The ProEmpower solutions enabled the collection of clinical parameters by the patient, using a smartphone integrated with medical devices. The data collected by the integrated devices (glucometer, sphygmomanometer, scale, smart watch for heart rate monitoring, and step counter) were automatically sent to the shared care plan.	Mobile-based/mobile app (integrated with medical devices such as glucometer, sphygmomanometer, scale, smart watch for heart rate monitoring, and step counter)	EG: At baseline and after an average follow-up of 8 months, glycosylated hemoglobin, body weight, blood pressure, and blood lipids were measured in the experimental group using the ProEmpower solutions. CG: Participants randomized to this group did not receive the ProEmpower mobile-based intervention. They continued with their pre-study habits and served as a comparison group for the analysis of outcomes.	No	8 months	8 months	Not reported	The pandemic restrictions affected the completeness of the data (follow-up visits and scheduled measurement)
Hesseldal at al (2022) [56]	Digital coaching intervention: initial 1 h face-to-face motivational interview followed by digital coaching using behavioral change techniques enabled by individual live monitoring.	Mobile-based and virtual coaching/mobile app	EG: Usual care plus digital lifestyle coaching. After the initial interview from the health care professionals, they received the health coach weekly (asynchronous digital coaching for each participant) that included inspiring them, commending them on goal attainment, and seeking to help them stay motivated. The subsequent asynchronous eHealth coaching sessions were carried out once a week for the first 6 months and then once a month for the last 6 months, as maintenance. CG: They received only the usual care preferred by the patient and their doctor.	No	12 months	6 and 12 months	Not reported	Many of the dropouts occurred at random due to COVID-19 restrictions; this may explain the nonsignificant effect of the intervention on HbA1c
Kim et al. (2024) [67]	Physical activity encouragement intervention based on a smartphone personal health record (PHR) application on step count increases, glycemic control, and body weight.	Mobile-based/mobile app with step count	CG: Used a smartphone PHR app. EG: Used the app and received individualized motivational text messages (intervention group) to increase daily steps.	No	3 months	3 and 6 months	Not reported	N/A
Lim et al. (2021) [59]	Intervention through the nBuddy Diabetes mobile app and educated to self-monitor their weight, diet, physical activity, and blood glucose levels for 6 months.	Mobile-based/mobile app	EG: At baseline received standard face-to-face dietary advice from a dietitian, were provided with a digital weighing scale, and were encouraged to 150 min per week of moderate intensity PA. They used the Nutritionist Buddy Diabetes mobile app that includes goal–setting, stimulus control, problem solving, self-monitoring their diet, PA, weight and blood glucose levels, cognitive restructuring, and motivational interviewing. CG: At baseline, received standard face-to-face dietary advice from a dietitian, were provided with a digital weighing scale, and were encouraged to 150 min per week of moderate intensity PA.	No	6 months	3 and 6 months	Median overall app utilization in the intervention group was 97.8% during the first 3 months and 91.7% during 4 to 6 months of the intervention period. The average two-way dietitian-to-participant interactions via the app’s chat function were 3 days per week in the first 3 months, and 2 days per week in the subsequent 3 months.	N/A
Moravcová et al. (2024) [61]	Comparison between the effects of an intensive in-person weight loss intervention program and Vitadio digital therapy (e-health).	Mobile-based and virtual coaching/mobile app	EG: Intervention through Vitadio, which is a certified class I medical device designed to support diabetes patients in making healthy lifestyle choices and improving their self-management, consisting of a 3-month intensive phase followed by a sustaining phase. The application uses a series of personalized daily tasks and automated messages to help patients establish a new, healthy routine. CG: This group was offered access to five in-person lifestyle consultations with a physician, dietitian, and/or educational nurse with a nutrition background from the Department of Exercise Medicine and Cardiovascular Rehabilitation.	Yes	6 months	3 and 6 months	Not reported	Plans to extend the study to evaluate the durability of these effects were hindered by high attrition rates following the intervention period due to the COVID-19 pandemic, which created significant obstacles for RCTs requiring in-person clinical assessments in hospital settings
Wang et al. (2018) [57]	Behavioral lifestyle intervention using mobile or paper-based tools for self-monitoring.	Mobile health-based self-monitoring and online telehealth/smartphone and mobile app and devices (pedometers, weight scales, and food scales)	EG: Divided into 2 groups (mobile group and paper group) received a standard behavioral lifestyle intervention comprising 11 group sessions—weekly for month 1, biweekly for months 2 and 3, and monthly for months 4 to 6, and an individual session after month 3. Participants received training on how to self-monitor their diet and exercise habits, weight, and blood glucose in the first two sessions. Both groups were instructed to record their exercise activities (minutes and type), specify the foods they ate (amount, number of calories, fat grams, and carbohydrates), their weight, and their blood glucose using a paper diary or an electronic diary, depending on their group randomization. CG: Individual visits or interactive group classes. Patients were not asked to self-monitor diet, activity, and weight on a daily basis.	Yes	6 months	3 and 6 months	The median rate of session attendance at the 11 group sessions was 100% for the mobile group and 81.8% for the paper group. Mobile group: the median percentage of days with at least one self-monitoring entry for diet, PA, weight, and glucose was 96.6%, 37.3%, 49.7%, and 72.7%, respectively. The paper group was 8.1%, 1.2%, 2.5%, and 2.5%, respectively.	Rural area: none of the participants reported owning a smartphone
Whittemore et al. (2020) [64]	Intervention through the ¡Sí, yo puedo! program that incorporated relevant theoretical underpinnings, educational content, and interactive strategies of 4 evidence-based programs for Hispanic adults with T2D to meet the needs of adults with T2D with limited resources, expertise of providers, and the systems of care of the Seguro Popular clinics in Mexico City.	Mobile-based self-management, text/picture messages, and face-to-face visits	EG: Received standard T2D care at the Seguro Popular clinic as aforementioned. They also received the ¡Sí, Yo Puedo! program which was developed after formative research with adults with T2D in Mexico. The program included 7 interactive group-based educational sessions on diabetes self-management. The nutrition component was central in the delivery of the intervention. based on “the smart plate” (modified for T2D). Behavioral support was also provided in all sessions, weekly goals, phone calls every two weeks, and text/picture messages daily during the 6 months of intervention. CG: No mobile-based intervention was implemented with participants in the control group. They were placed on a waiting list and continued with their pre-study habits.	Yes	6 months	3 and 6 months	Attendance was high at 89% across all sessions, and attrition was low at 6.4% (n = 3) at 6 mo. A total of 96% of participants received the text at 3 months and 100% at 6 months, and for picture messages, 83% at 3 months and 88% at 6 months. Adherence to protocol implementation was high, with goals and objectives completely fulfilled in 91% of the sessions and mostly achieved in 7% of sessions.	N/A
Yin et al. (2022) [58]	Telemedicine	Mobile health-based self-monitoring and online telehealth/mobile app and device (glucometers)	EG: They were followed up four times a week in the first 3 months and twice a week in the next 3 months. Doctors used reminders for diet guidance and exercise advice, including energy intake and food exchange methods. They uploaded their daily dietary intake on the telemedicine app. Additionally, the app recorded the patients’ daily steps and automatically transferred them to the medical server. Further, exercise guidance was provided to each patient. Blood glucose levels were monitored using a glucometer and were automatically transferred to the hospital telemedicine app. CG: They were followed up through conventional outpatient clinic appointments every 2 weeks, and telephone follow-up was used during the isolation period for the glucose data management. Traditional health education, which included diet, exercise, and medication guidance, was provided during clinic visits.	No	6 months	21 days, 3 months, and 6 months	Not reported	In the framework of the COVID-19 disease, all patients underwent an initial physical examination and blood sample collection, followed by a mandatory home quarantine for 21 days

CG = control group, EG = experimental group, HbA1c = glycosylated hemoglobin, PA = physical activity, T2D = type 2 diabetes, IVR = interactive voice response, BCTs = behavioral change techniques. * Hybrid intervention = technology-based intervention combined with conventional care.

### 3.3. Risks of Bias in Included Studies

**Figure 2:**
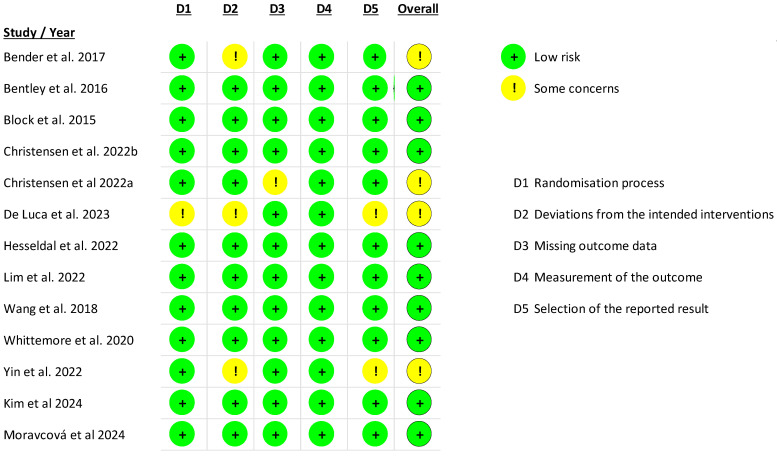
An assessment of the risk of bias of the included studies [54,55,56,57,58,59,61,62,63,64,65,66,67].

The included studies were RCTs in which the intervention was delivered through a mobile application to analyze the impact on various outcomes. Four studies [54,55,58,63] demonstrated some concerns regarding the risks of bias. For the remaining nine studies [56,57,59,61,62,64,65,66,67], no risks of bias were detected across the five analyzed domains (Figure 2).

### 3.4. Effects of the Interventions

The following meta-analysis was conducted to examine the impact of mHealth interventions on individuals with concurrent T2D and overweight or obesity. In all cases, the pre-post intervention change data are reported. Subgroup analyses based on the duration of the intervention were conducted to provide more detailed insights for each outcome.

#### 3.4.1. Changes in Hb1Ac (%)

**Figure 3:**
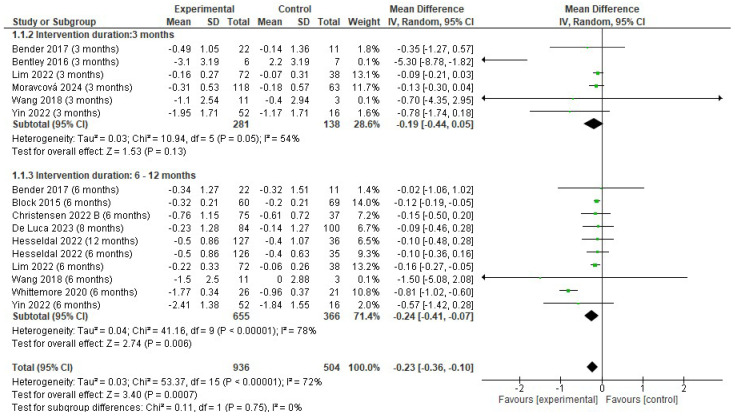
Forest plot of meta-analysis results in changes in percentage of Hb1Ac. CI, confidence interval. ◆ Black Diamonds means the pooled effect size from the meta-analysis (the width of the diamond represents the 95% CI) [54,56,57,58,59,61,62,63,64,65,66].

There was a reduction in HbA1c (%) favoring the experimental group (EG) (MD −0.23; 95% CI −0.36 to −0.10; *p* < 0.001; I^2^ = 72%). The reduction in HbA1c (%) was lower in the EG at 3 months (MD −0.19; 95% CI −0.44 to 0.05; *p* = 0.13; I^2^ = 54%) and 6 to 12 months (MD −0.24; 95% CI −0.41 to −0.07; *p* = 0.006; I^2^ = 78%) (Figure 3).

#### 3.4.2. Changes in Body Weight

**Figure 4:**
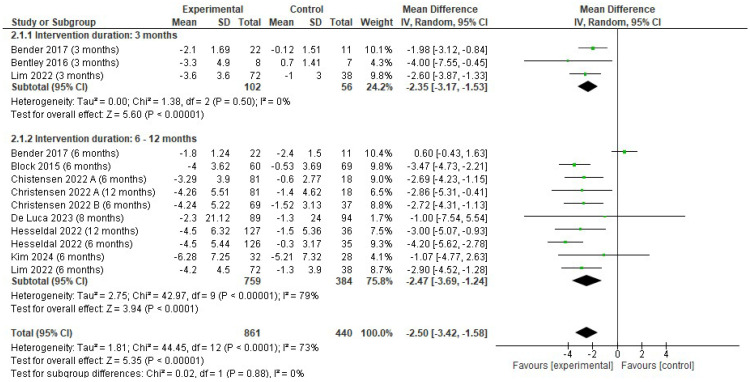
Forest plot of meta-analysis results in changes in body weight (kg). CI, confidence interval. ◆ Black Diamonds means the pooled effect size from the meta-analysis (the width of the diamond represents the 95% CI) [54,55,56,59,62,63,65,66,67].

There was a reduction in body weight favoring the EG (MD −2.50; 95% CI −3.42 to −1.58; *p* < 0.001; I^2^ = 73%). The reduction in body weight in the EG was higher at 6–12 months (MD −2.47; 95% CI −3.69 to −1.24; *p* < 0.001; I^2^ = 79%) than at 3 months (MD −2.35; 95% CI −3.17 to −1.53; *p* < 0.001; I^2^ = 0%) (Figure 4).

#### 3.4.3. Changes in Triglycerides

**Figure 5:**
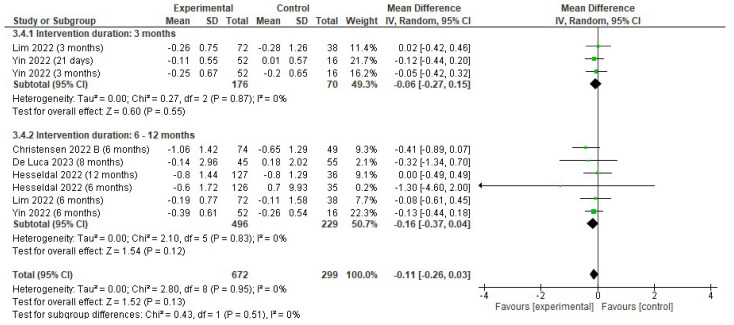
Forest plot of meta-analysis results in changes in triglycerides. CI, confidence interval. ◆ Black Diamonds means the pooled effect size from the meta-analysis (the width of the diamond represents the 95% CI) [56,58,59,63,65].

Non-significant effects on triglycerides (mmol/L) were found (MD −0.11; 95% CI −0.26 to 0; *p* = 0.13; I^2^ = 0%). Subgroup analyses showed a trend for the reduction in triglycerides in the EG to be larger at longer-term follow-up (i.e., 6–12 months; MD −0.16; 95% CI −0.37 to 0.04; *p* = 0.12; I^2^ = 0%) than at 3 months (MD −0.06; 95% CI −0.27 to 0.15; *p* = 0.55; I^2^ = 0%), but both effects were not significant (Figure 5).

#### 3.4.4. Changes in Cholesterol, LDL, and HDL

**Figure 6:**
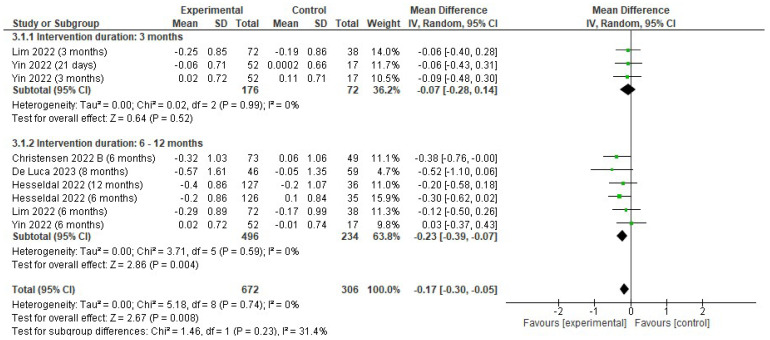
Forest plot of meta-analysis results in changes in total cholesterol. CI, confidence interval. ◆ Black Diamonds means the pooled effect size from the meta-analysis (the width of the diamond represents the 95% CI) [56,58,59,63,65].

There was a reduction in total cholesterol (mmol/L) favoring the EG (MD −0.17; 95% CI −0.30 to −0.05; *p* = 0.008; I^2^ = 0%). Subgroup analyses showed that the effects in total cholesterol (mmol/L) were significant in the EG at 6–12 months (MD −0.23; 95% CI −0.39 to −0.07; *p* = 0.004; I^2^ = 0%) but not at 3 months (MD −0.07; 95% CI −0.28 to 0.14; *p* = 0.52; I^2^ = 0%) (Figure 6).

**Figure 7:**
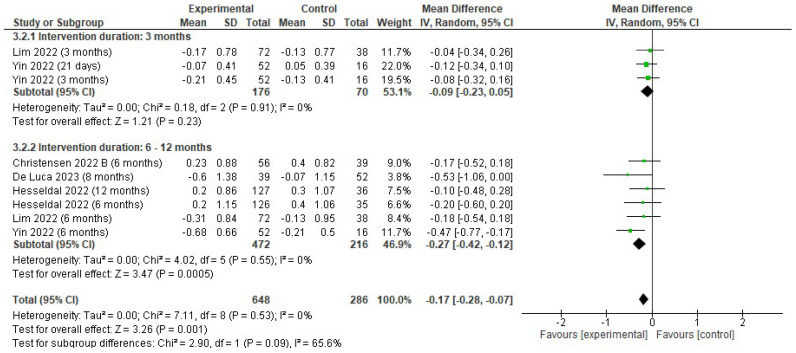
Forest plot of meta-analysis results in changes in LDL. CI, confidence interval. ◆ Black Diamonds means the pooled effect size from the meta-analysis (the width of the diamond represents the 95% CI) [56,58,59,63,65].

There was a reduction in LDL (mmol/L) favoring the EG (MD −0.17; 95% CI −0.28 to −0.07; *p* = 0.001; I^2^ = 0%). Subgroup analyses showed that the effects on LDL (mmol/L) were significant in the EG at 6–12 months (MD −0.27; CI −0.42 to −0.12; *p* < 0.001; I^2^ = 0%) but not at 3 months (MD −0.09; CI −0.23 to 0.05; *p* = 0.23; I^2^ = 0%) (Figure 7).

**Figure 8:**
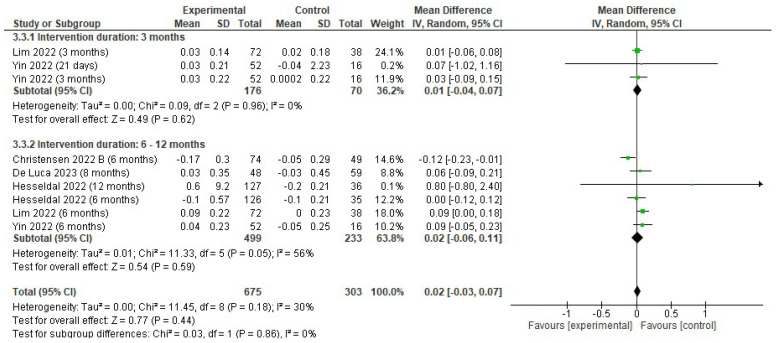
Forest plot of meta-analysis results in changes in HDL. CI, confidence interval. ◆ Black Diamonds means the pooled effect size from the meta-analysis (the width of the diamond represents the 95% CI) [56,58,59,63,65].

There was a non-significant effect on HDL (mmol/L) (MD 0.02; 95% CI −0.03 to 0.07; *p* = 0.44; I^2^ = 30%). Non-significant effects on HDL (mmol/L) were observed in both trials lasting 3 months (MD 0.01; 95% CI −0.04 to 0.07; *p* = 0,62; I^2^ = 0%) and in trials lasting 6 to 12 months (MD 0.02; 95% CI −0.06 to 0.11; *p* = 0.59; I^2^ = 56%) (Figure 8).

## 4. Discussion

The objective of this study was to examine the effect of mHealth interventions on individuals with concurrent T2D and overweight or obesity, through a systematic review and meta-analysis. Understanding these potential differences is crucial for tailoring effective treatment strategies for this population, as the interplay between T2D and obesity might influence the effectiveness of mHealth interventions. Significant conclusions were drawn when subgroups were analyzed based on the duration of the intervention. For every outcome, two subgroups were created (3 months and 6–12 months). The findings indicate that mHealth interventions significantly reduced Hb1Ac levels compared to control groups, with more substantial effects observed in longer-duration interventions (6–12 months). Likewise, significant weight, total cholesterol, and LDL reductions were achieved, particularly in interventions lasting 6–12 months, showing greater reductions than shorter-term interventions. High adherence rates were reported in interventions involving mobile apps and wearables, which are crucial for longer interventions (6–12 months) to yield better outcomes and contribute to achieving these results.

### 4.1. Effects on Hb1Ac

Several preview reviews [68,69,70,71,72,73] that deal with mHealth interventions through apps or wearables for T2D patients have shown effectiveness for the reduction in the percentage of Hb1Ac throughout the interventions. Eberle et al. [68] revealed an effect size of −0.54%, 95% CI −0.8 to −0.28 when compared to usual care. Similarly, Hou et al. [69], in a review of 14 studies, reported a reduction in Hb1Ac of −0.49%, 95% Cl −0.68 to −0.30; I^2^ = 10% among participants using mobile apps compared to usual care, which was associated with a moderate grade of evidence. Timpel et al. [70] reported that mobile app interventions in populations with T2D resulted in a reduction in HbA1c of −0.33%, 95% CI −0.59 to −0.06; I^2^ = 70%. Another study [71] found that in 10 out of 12 studies analyzed, the mHealth intervention group had an HbA1c reduction greater than 0.3% compared to the comparison group. Verma et al. [72] observed a decrease in Hb1Ac by −0.44%, 95% CI −0.79 to 0.10, *p* = 0.01, I^2^ = 87%. Anderson et al. [73] showed that telehealth interventions, when pooled across studies with a sample comprising more than 50% Black and Hispanic participants, led to a reduction in HbA1c of −0.46%, 95% CI −0.64 to −0.28.

The results of our review indicate a slightly lower effect on HbA1c (%) reduction compared to previous studies (MD of −0.23, 95% CI −0.36 to −0.10). A possible reason for this reduced effect could be the presence of both obesity and diabetes in the participants studied. The combination of severe insulin resistance, chronic low-grade inflammation, and visceral adiposity—commonly seen in individuals with both conditions—may diminish the effectiveness of mHealth interventions in improving glycemic control compared to patients with diabetes alone [74]. Visceral fat accumulation in T2D patients negatively impacts glycemic control through decreased peripheral insulin sensitivity and enhanced gluconeogenesis [75]. The increase in adipose tissue has been related to the increase in the production of proinflammatory cytokines, which, together with fatty acids, seem to be responsible for the development of insulin resistance [76]. This greater inflammation can interfere with insulin signaling and metabolic function [77], which could reduce the effectiveness of exercise in glucose control [78] Although most studies suggest that exercise improves glycemic control, insulin sensitivity, and hormonal responses in both lean patients and patients with obesity, other studies indicate that obesity can alter these responses, reducing the efficacy of exercise in improving glucose regulation [79].

### 4.2. Effects on Body Weight

Overweight and obesity have been largely studied and treated using mHealth over the past decades. Several studies have demonstrated the effectiveness and efficacy of the use of mobile phone apps in managing body weight loss compared to patients who did not use these tools [80,81,82,83,84,85]. Antoun et al. [80] reported a significant body weight loss at 3 months (MD −1.99 kg, 95% CI −2.19 to −1.79; I^2^ = 81%) and 6 months (MD −2.80 kg, 95% CI −3.03 to −2.56 kg; I^2^ = 90%). Additionally, a meta-analysis [81] revealed the significant effects of interventions on weight loss at 3 months (MD −2.18 kg, 95% CI −3.59 to −0.78; I^2^ = 87%) and at 6 months (MD −2.15, 95% CI −3.25 to −1.05; I^2^ = 52%) and at 9 to 12 months (MD −1.63 kg, 95% CI −2.99 to −0.26; I^2^ = 0%), concluding that weight reduction was most pronounced at 3 months but tapered down after 12 months. In the current review, significant effects of interventions on weight loss were observed at 3 months (MD −2.35 kg, 95% CI −3.17 to −1.53) and at 6 to 12 months (MD −2.47 kg, 95% CI −3.69 to −1.24).

Additionally, the lower physical capacity and potential comorbidities in these patients might limit the intensity and adherence to interventions [85]. The development of new methods to facilitate patients’ increased physical activity and the long-term maintenance of physical activity is fundamental for the maintenance of weight loss and for reducing the health risk of individuals with obesity [86]. Self-monitoring is the cornerstone of the behavioral treatment of obesity. The greater the use of self-monitoring, the greater the weight loss [87]. The latter approach attributes the inactivity and high dropout to limited discretionary time and the slow accrual of visible benefits. However, a higher intensity of physical activity has been associated with non-adherence and dropout, whereas a longer duration has not [88].

Most studies on app-based interventions have durations ranging from 12 weeks to 6 months, with some extending up to 24 months [89,90,91]. Longer intervention periods (≥6 months) are associated with more sustained weight loss and better glycemic control [32,91]. In the current review, reductions in weight in the long term were greater than in the short term. Long-term interventions can yield positive results, but can struggle to maintain the required levels of adherence and engagement. Future research should focus on longer-term studies with larger sample sizes to better understand the optimal duration and sustainability of these interventions.

### 4.3. Effects on Lipid Profiles: Triglycerides, Cholesterol, LDL, and HDL

Findings for lipid profiles in our review generally align with other reviews. Akbari et al. [92] indicated that mHealth interventions significantly decreased total (SMD −0.54, 95% CI −1.05 to −0.03) and LDL-cholesterol levels (SMD −0.66, 95% CI −1.18 to −0.15) and had a non-significant effect on triglycerides (SMD −0.14, 95% CI −0.56 to 0.28) and HDL-cholesterol levels (SMD −0.35, 95% CI −0.81 to 0.11). Another review [93] with T2D patients comparing different types of telemedicine interventions showed non-significant effects on HDL-cholesterol (0.01 mmol/L, 95% CI −0.03 to 0.05), LDL-cholesterol (0.08 mmol/L, 95% CI −0.22 to 0.37), triglyceride (−0.08 mmol/L, 95% CI −0.31 to 0.15), and total cholesterol (−0.10 mmol/L, 95% CI −0.25 to 0.04) levels. In a further review [44], three studies that measured serum lipids outcomes were included, and there was no significant difference between the EG and CG (LDL-c: −0.12 mmol/L, 95% CI −0.34 to 0.11 mmol/L, *p* = 0.30; HDL-c: 0.01 mmol/L, 95% CI −0.05 to 0.07 mmol/L, *p* = 0.81; TG: −0.06 mmol/L, 95% CI −0.32 to 0.19 mmol/L, *p* = 0.62 and TC: −0.15 mmol/L, 95% CI −0.6 to 0.3 mmol/L, *p* = 0.50).

Another review [94] found that a combined intervention of self-monitoring apps with health coaching in people with overweight and obesity significantly improved triglyceride levels by 0.22 mg/dL, 95% CI −0.33 mg/dL to −0.11 mg/dL, *p* = 0.008; I^2^ = 0% but not total cholesterol levels.

In the current review, a non-significant effect was observed on HDL (mmol/L) (MD 0.02, 95% CI −0.03 to 0.07; *p* = 0.44; I^2^ = 30%) and TG (MD −0.11, 95% CI −0.26 to 0.03; *p* = 0.13; I^2^ = 0%). However, the significant effects were observed when trial durations were longer (6–12 months). In these cases, significant effects were observed in total cholesterol (MD −0.23, 95% CI −0.39 to −0.07; *p* = 0.004; I^2^ = 0%) and LDL (MD −0.27, CI −0.42 to −0.12; *p* = 0.0005; I^2^ = 0%) but still not in triglycerides (MD −0.16, 95% CI −0.37 to 0.04; *p* = 0.12; I^2^ = 0%). These findings suggest that the duration of the interventions may be key to obtaining a significant improvement in lipid profiles, and it is necessary to design strategies that support the maintenance of these effects in the long term.

### 4.4. Adherence to mHealth Interventions

MHealth tools show potential in improving patient adherence to chronic disease management, though current evidence on their effectiveness is mixed [95]. Adherence to mHealth programs in the studies included in our review varied based on factors such as the duration of the intervention and the use of wearable devices. For studies combining wearable devices with mobile apps [54,66] adherence was higher in the 6-month intervention compared to the 3-month program. Specifically, in the 6-month intervention, adherence rates were as follows: attendance at visits (CG 100%, EG 95%) and Fitbit usage (EG 97%, CG 91%). In the 3-month intervention, adherence rates were as follows: week 1–6 (62%), week 7–12 (65%), and week 13–16 (extended weight maintenance period) (75%). These findings suggest that longer interventions may improve adherence, though more comparative studies are needed to confirm this. Furthermore, this idea is aligned with the majority of studies [32,96] that recommend a duration of at least 6 months for mHealth programs to manage T2D and overweight or obesity.

Other studies [57,59,62,64] included in our review used only mobile apps for their interventions. In these cases, the duration of the intervention was 6 months, and adherence to programs was also high. This can be due to the association of overweight and T2D, as persistence to treatment is usually high [97]. Notably, adherence to self-monitoring was higher when mobile apps were used. One study [64] compared adherence to a self-monitoring diet, PA, weight, and glucose when using or not using a mobile app. The results showed higher adherence rates with app use (diet: 96.6% vs. 8.1%; PA: 37.3% vs. 1.2%; weight 49.7% vs. 2.5%; glucose 72.7% vs. 2.5%). These findings underscore the benefits of mobile apps in facilitating the self-monitoring of T2D and overweight or obesity.

### 4.5. Limitations

It is worth highlighting the variety of interventions of the studies analyzed, mainly related to the differences between experimental and control group types of treatment. The effectiveness of these interventions is strongly linked to user awareness, education, and the behavior change communication methods employed. However, these studies often lack details on how physical activity is managed within the interventions. Aspects such as the intervention design, the specific mobile application used, and how physical activity, dietary habits, or education are incorporated are crucial factors influencing the outcomes. This makes it difficult to analyze the influence of each intervention more accurately on the results of the different outcomes, and thus they should be interpreted with caution.

A second limitation can be attributed to the differences that could exist between the usual care from the local medicine in different countries that are included in our study, which may impact the results of the outcomes measured.

It should also be noted that the sample sizes of the included studies were indeed highly variable. Recruitment and retention were particularly challenging across studies, as participants needed to meet both clinical criteria and possess the necessary technological skills. Additionally, in certain studies, adherence was negatively impacted by the requirement to wear portable devices over extended periods, further affecting retention rates.

Lastly, baseline levels of weight, BMI, and Hb1Ac are different when comparing the included studies in our review. Although our study analyzed the changes between baseline and final values, this aspect must be taken into consideration.

## 5. Conclusions

This systematic review and meta-analysis found that mHealth interventions for individuals with concurrent T2D and overweight or obesity showed significant improvements in glycemic control, body weight, total cholesterol, and LDL, particularly for interventions lasting 6–12 months, with interventions incorporating a wearable achieving higher adherence. Taken together, these findings highlight the considerable potential of mHealth tools for managing these complex and concurrent conditions.

The results from this systematic review and meta-analysis suggest an important clinical implication of mHealth tools as an effective and scalable solution for managing concurrent T2D and obesity, addressing the need for personalized, accessible and ongoing support.

Future research focused on optimizing long-term engagement and outcomes in mHealth interventions for patients with concurrent T2D and obesity is needed to maximize the real-world impact of these interventions.

## Figures and Tables

**Table 1:** GRADE quality of evidence.

Certainty Assessment							№ of Patients		Effect		Certainty	Importance
№ of Studies	Study Design	Risk of Bias	Inconsistency	Indirectness	Imprecision	Other Considerations	mHealth	[Usual Care]	Relative (95% CI)	Absolute (95% CI)		
Glycated Hemoglobin (HbA1c)												
11	randomized trials	not serious ^a^	very serious ^b^	serious ^c^	not serious ^d^	very strong association	936/1440 (65.0%)	504/1440 (35.0%)	not estimable	2 more per 1000 (from 1 more to 4 more)	⨁⨁⨁◯ Moderate ^a,b,c,d^	CRITICAL
Weight												
9	randomized trials	not serious ^a^	very serious ^e^	serious ^c^	not serious ^f^	very strong association	861/1301 (66.2%)	440/1301 (33.8%)	not estimable	25 more per 1000 (from 16 more to 34 more)	⨁⨁⨁◯ Moderate ^a,c,e,f^	CRITICAL

Abbreviations: CI, confidence interval. ^a^ The assessment of risks of bias using the RoB 2.0 tool. ^b^ Considerable heterogeneity was observed across studies (I^2^ = 72%), indicating substantial inconsistency in the results. ^c^ While the population across studies is consistent, the interventions encompass different types of mHealth approaches, and comparators vary between studies. This variation introduces serious indirectness, potentially limiting the direct applicability and comparability of results. ^d^ CI [−0.23, −0.10]. ^e^ Considerable heterogeneity was observed across studies (I^2^ = 73%), indicating substantial inconsistency in the results. ^f^ CI [−0.36, −0.10].

## Data Availability

The data confirming the results obtained are available through the corresponding author.

## Supplementary Materials

The following supporting information can be downloaded at https://www.mdpi.com/article/10.3390/jfmk10030292/s1, Table S1: PRISMA Checklist; Supplementary File S1: Research Strategy.

## Abbreviations

BMI	Body Mass Index
DM	Diabetes Mellitus
Hb1Ac	Glycosylated Hemoglobin
HDL	High-Density Lipoprotein
LDL	Low-Density Lipoprotein
PA	Physical Activity
RCTs	Randomized Controlled Trials
TG	Triglycerides
T2D	Type 2 Diabetes
