# Supplementary material for: Mobile Health Interventions for Individuals with Type 2 Diabetes and Overweight or Obesity—A Systematic Review and Meta-Analysis
Source: J Funct Morphol Kinesiol. 2025 Jul 29;10(3):292. doi: 10.3390/jfmk10030292 (PMC12372011; doi:10.3390/jfmk10030292)
Supplement: Supplementary file 1 [file jfmk-10-00292-s001.zip › File S1. Research Strategy.pdf]

Supplementary File S1. Research strategy.

Date of Search: 2025-07-09

Field Tags: abstract

Publication years: 2010/01/01 to 2025/07/09

Filter: randomized controlled trial

Number of total hits:

PUBMED

Number of hits: 385

(((("diabete"[All Fields] OR "diabetes mellitus"[MeSH Terms] OR ("diabetes"[All Fields] AND "mellitus"[All Fields]) OR "diabetes mellitus"[All Fields] OR "diabetes"[All Fields] OR "diabetes insipidus"[MeSH Terms] OR ("diabetes"[All Fields] AND "insipidus"[All Fields]) OR "diabetes insipidus"[All Fields] OR "diabetic"[All Fields] OR "diabetics"[All Fields] OR "diabets"[All Fields]) AND ("obeses"[All Fields] OR "obesity"[MeSH Terms] OR "obesity"[All Fields] OR "obese"[All Fields] OR "obesities"[All Fields] OR "obesity s"[All Fields])) OR ("overweight"[MeSH Terms] OR "overweight"[All Fields] OR "overweighted"[All Fields] OR "overweightness"[All Fields] OR "overweights"[All Fields]) OR ("metabolic syndrome"[MeSH Terms] OR ("metabolic"[All Fields] AND "syndrome"[All Fields]) OR "metabolic syndrome"[All Fields])) AND ("mhealth s"[All Fields] OR "telemedicine"[MeSH Terms] OR "telemedicine"[All Fields] OR "mhealth"[All Fields] OR "wearable"[All Fields] OR (("appl plant sci"[Journal] OR "apps"[All Fields]) AND ("intervention s"[All Fields] OR "interventions"[All Fields] OR "interventive"[All Fields] OR "methods"[MeSH Terms] OR "methods"[All Fields] OR "intervention"[All Fields] OR "interventional"[All Fields])) OR ("mobile applications"[MeSH Terms] OR ("mobile"[All Fields] AND "applications"[All Fields]) OR "mobile applications"[All Fields] OR ("mobile"[All Fields] AND "apps"[All Fields]) OR "mobile apps"[All Fields])) AND "hasabstract"[All Fields] AND ("hasabstract"[All Fields] AND 2010/01/01:2024/07/29[Date - Publication]) AND "randomized controlled trial"[Publication Type]) AND ((fha[Filter]) AND (randomizedcontrolledtrial[Filter]))

Translations

randomizedcontrolledtrial[Filter]: randomized controlled trial [PT]

SCOPUS

Date of search: 2025/07/09

Field Tags: Tittle-abs-key

Publication years: 2010 to date

Number of Hits: 703

(TITLE-ABS-KEY
(diabetes AND obesity OR overweight OR metabolic syndrome) AND (mhealth OR weara
ble OR apps interventions OR mobile apps) AND PUBYEAR > 2009 AND PUBYEAR
< 2026)

WEB OF SCIENCE

Database: All Databases

Searches:

1: diabetes (Topic) AND obesity (Topic) OR overweight (Topic) - Results: 442016

2: mhealth (Topic) OR apps interventions (Topic) OR mobile apps (Topic) OR wearable (Topic) - Results: 179333

3: #1 AND #2 - Results: 1911

4: #1 AND #2 and Article or Clinical Trial (Document Types)

Date Run: Wed Jul 09 2025

Results: 1104
